# Estimating Grizzly and Black Bear Population Abundance and Trend in Banff National Park Using Noninvasive Genetic Sampling

**DOI:** 10.1371/journal.pone.0034777

**Published:** 2012-05-02

**Authors:** Michael A. Sawaya, Jeffrey B. Stetz, Anthony P. Clevenger, Michael L. Gibeau, Steven T. Kalinowski

**Affiliations:** 1 Western Transportation Institute, Montana State University, Bozeman, Montana, United States of America; 2 Department of Ecology, Montana State University, Bozeman, Montana, United States of America; 3 Sinopah Wildlife Research Associates, Missoula, Montana, United States of America; 4 Mountain National Parks, Parks Canada, Lake Louise, Alberta, Canada; University of Manitoba, Canada

## Abstract

We evaluated the potential of two noninvasive genetic sampling methods, hair traps and bear rub surveys, to estimate population abundance and trend of grizzly (*Ursus arctos*) and black bear (*U. americanus*) populations in Banff National Park, Alberta, Canada. Using Huggins closed population mark-recapture models, we obtained the first precise abundance estimates for grizzly bears (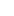 = 73.5, 95% CI = 64–94 in 2006; 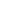 = 50.4, 95% CI = 49–59 in 2008) and black bears (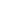 = 62.6, 95% CI = 51–89 in 2006; 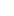 = 81.8, 95% CI = 72–102 in 2008) in the Bow Valley. Hair traps had high detection rates for female grizzlies, and male and female black bears, but extremely low detection rates for male grizzlies. Conversely, bear rubs had high detection rates for male and female grizzlies, but low rates for black bears. We estimated realized population growth rates, lambda, for grizzly bear males (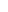 = 0.93, 95% CI = 0.74–1.17) and females (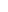 = 0.90, 95% CI = 0.67–1.20) using Pradel open population models with three years of bear rub data. Lambda estimates are supported by abundance estimates from combined hair trap/bear rub closed population models and are consistent with a system that is likely driven by high levels of human-caused mortality. Our results suggest that bear rub surveys would provide an efficient and powerful means to inventory and monitor grizzly bear populations in the Central Canadian Rocky Mountains.

## Introduction

Carnivore populations are disappearing globally at an incredible rate due to anthropogenic causes [1], but dedicated monitoring programs rarely exist due to technical, financial, and logistical constraints [2]. Conservationists have long predicted that major shifts in ecosystems can follow changes in the abundance and distribution of apex consumers (e.g. carnivores), but a recent review of empirical evidence sheds new light on the critical role that consumers play to ensure the maintenance of top-down ecological processes at every trophic level [1]. Climate change further affects the removal of top-down selective pressures [3] and makes it increasingly important for conservation managers to be able to monitor changes in carnivore populations and respond to biodiversity threats accordingly [4]. Reliable estimates of population parameters such as abundance and population growth rate are needed for successful adaptive management of carnivore species, but these data are difficult to collect for secretive and wide-ranging species that often occur at low densities such as grizzly (*Ursus arctos*) and black bears (*U. americanus*) [5].

Advancements in molecular genetic analysis techniques provide new cost-effective and reliable monitoring methods that do not require capturing or handling animals [2]. DNA extracted from hair and scat samples collected noninvasively can be used to identify species, individuals, and their sex. Noninvasive genetic sampling (NGS) provides an alternative to conventional bear population monitoring methods such as biomarking and radio-telemetry [6]. Genetic monitoring can provide information on abundance, distribution, vital rates, and genetic interchange, but wildlife managers have at times been reluctant to embrace NGS methods because they are relatively new [7], [8]. Understandably, there has been concern that genotyping errors might inflate DNA-based abundance estimates, but recent studies show that NGS methods can provide reliable and accurate information on carnivore populations [9]. Barbed wire hair traps developed by Woods et al. [10] have become the standard for collecting genetic data to estimate bear population parameters in North America [11], [12]; however, the relative strengths and weaknesses of alternative NGS monitoring methods such as surveys using bear rubs (i.e. trees, power poles, etc) must be evaluated before one method becomes well-established.

In the past 15 years, there has been a dramatic increase in research on North American bear populations using NGS [12], but other than one study which used scat detection dogs with limited success [13], research has almost exclusively focused on hair traps as the single DNA collection method. Numerous studies throughout North America (e.g. [14]–[16]) have used barbed wire hair traps in a mark-recapture framework to estimate population abundance and density for grizzly and black bears. Research conducted in the Northern Continental Divide Ecosystem of Montana has shown that bear rubs detect many grizzly bears not detected in hair traps and the use of bear rubs significantly improves precision of grizzly bear abundance estimates compared to estimates with hair traps alone [14], [15], [17]. Stetz et al. [18] used simulations based on empirical data from the Northern Continental Divide Ecosystem to demonstrate that bear rub surveys can produce precise estimates of grizzly bear population growth rate with just several years of sampling, however, there has yet to be a published study that has used bear rub surveys to directly estimate abundance or population growth rate.

Precise and unbiased estimates of carnivore population abundance and 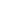 are especially important to adaptive management in areas with low densities and limited reproductive capacity such as Banff National Park (BNP) in Alberta, Canada which is home to one of the lowest density and slowest reproducing populations of grizzly bears in North America [19]. Differences in density and population growth rates could greatly affect the performance of population monitoring methods; therefore sampling methods must be evaluated in different geographic areas in order to gain a comprehensive understanding of their ability to monitor wildlife populations under variable environmental and demographic conditions. BNP provides a fitting contrast to the Northern Continental Divide Ecosystem in MT, the central area of focus for past research using bear rubs [15].

Population monitoring is particularly important in national parks such as BNP because protected areas often serve as source populations for much larger geographic areas [20]. BNP along with Yoho and Kootenay National Parks comprise the UNESCO Rocky Mountain World Heritage Site and make up the core of the Central Canadian Rocky Mountains. With over four million visitors per year, however, BNP is also one of the world’s most heavily visited national parks and this high level of human visitation acts as a major stressor on the ecosystem [21]. BNP has identified grizzly bears as one of the park’s indicators of ecological integrity [22], but despite the protections afforded by the national park system and provincial parks in the region, much of the habitat within the ecosystem, including BNP, is unsecure for grizzly bears [23]. Studies have shown that human-caused mortality (e.g. highway and railway deaths) significantly affects grizzly and black bear survival in BNP [19], [24]. In June 2010, the Alberta government listed the grizzly bear as “threatened” under Alberta’s Wildlife Act based on over a decade of radio-telemetry research and five years of DNA-based population surveys [25]. This designation increased the prominence of BNP for grizzly bears in Alberta and added urgency to wildlife managers’ need for cost-effective techniques to inventory and monitor bear populations.

To successfully manage sympatric grizzly and black bear populations in the face of increasing human pressures, bear managers should understand the specific performance of hair traps and bear rubs to detect individuals and estimate demographic parameters for both species. In many areas of North America, grizzly and black bear diets completely overlap which may lead to direct competition between species [26]. Intraspecific competition could strongly influence the effectiveness of different survey methods, yet few studies (e.g. [27]) have compared NGS results from both species. NGS studies conducted in areas with sympatric populations readily collect hair from both grizzly and black bears and can produce demographic estimates for both species even though only one species, grizzly, is typically targeted. However, black bear hair is rarely analyzed in conjunction with grizzly bear hair due to financial constraints and less conservation concern for black bears in western North America [27].

The goal of this investigation was to evaluate the relative ability of two NGS methods, hair traps and bear rubs, to detect individuals and estimate population parameters for sympatric grizzly and black bear populations in the Bow Valley of BNP. Our specific objectives were to 1) estimate the abundance of grizzly and black bears in the Bow Valley of BNP using hair traps and bear rubs, 2) compare detection rates of hair traps and bear rubs for grizzly and black bears, and 3) evaluate the potential for using bear rubs for long-term monitoring of grizzly bear population growth rates in the Central Canadian Rocky Mountains. This is the first study to directly estimate abundance and population growth rates using bear rub surveys.

## Methods

### Ethics Statement

Our research did not involve capture or handling of animals, therefore did not require approval of animal care and use procedures. Permissions for field studies in BNP were given by Parks Canada Agency under research permit #BAN-2007-999. Permissions for field studies in Alberta provincial lands were given by the Alberta Minister of Community Development under research permit #RC-06-22.

### Study Area

**Figure 1:**
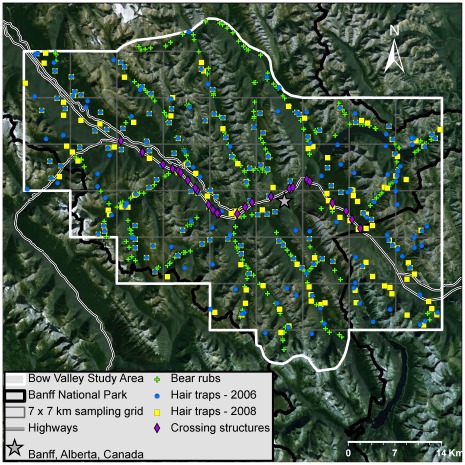
Noninvasive genetic sampling locations for grizzly and black bears in the Bow Valley Study Area of Banff National Park, Alberta, Canada. Locations of 420 hair traps, 321 bear rubs and 20 wildlife crossing structures, monitored between 21 April and 31 October of 2006–2008.

The flagship of Canada’s extensive national park system, BNP (6848 km^2^) in the Central Canadian Rocky Mountains of Alberta was established in 1885 making it the third national park created in the world. Our 2246 km^2^ Bow Valley Study Area (BVSA) was located approximately 120 km west of Calgary, Alberta, east of the Continental Divide in the central Rocky Mountains (Figure 1). The study area was mostly contained within BNP, but also extended slightly into Kootenay National Park and Alberta provincial lands. The lower Bow Valley is a human-dominated landscape with the Trans-Canada Highway, the Town of Banff with 8000 residents, a golf course, three ski areas, a railway, and a secondary highway. Between 1980 and 1998, 45 km of the Trans-Canada Highway were widened for safety reasons [28] and 25 wildlife crossing structures were constructed to reduce wildlife–vehicle collisions and facilitate wildlife movement across the roadway [29].

Detailed ecological descriptions of the study area can be found in Holroyd & Van Tighem [30] and Holland & Coen [31]. The lower slopes on both sides of the BVSA were dominated by lodgepole pine (*Pinus contorta*) forests on glacial till terraces while the dry, open south-facing slopes were dominated by Douglas fir (*Pseudotsuga menziesii*). Fluvial bottomlands were a mix of mature white spruce (*Picea glauca*), dry, open spruce forest, and moist shrubland [32]. Important foods for grizzly and black bears are bearberry (*Arctostaphylus uva-ursi*), horsetails (*Equisetum arvense*), graminoids, and buffaloberry (*Shepherdia canadensis*) [33], [34].

### Sampling

We initially defined our study area (Figure 1) as a 14 km buffer around the 45 km length of mitigated Trans-Canada Highway in BNP in order to assess the performance of wildlife crossing structures for grizzly and black bears in the Bow Valley [35]. We overlaid a 7×7 km grid, two cells deep, across our study area, for a total of 42 cells. Studies using hair traps for grizzly bear population estimates often employ a 7×7 km grid [36] to distribute effort across the region of interest. The orientation of our grid was chosen to be continuous with a larger grid used to inventory grizzly bears in Alberta [25]. We modified the study area boundary slightly to the north and south of the sampling grid to account for geographic features and minimize geographic closure violation.

We used two methods, hair traps and bear rubs, concurrently to collect hair from black and grizzly bears for genetic analysis. We surveyed bear rubs in 2006, 2007, and 2008. We deployed hair traps in 2006 and 2008, but chose not to use hair traps during our second field season due to the high cost and effort associated with hair trapping and the greater potential with repeated sampling for bears to show a negative trap response over time due to a waning interest in the scent lure [17], [37].

We placed one hair trap in each grid cell for each of five 14-day sampling periods from mid-May to mid-August in 2006 and 2008. Hair traps consisted of a 25 m length of barbed wire nailed at a height of 50 cm to a series of trees to form an enclosure [10]. We lured bears into the enclosure by placing 3 L of liquid scent lure poured on rotten wood and other debris piled in the center of the enclosure [15]. The scent lure consisted of a 2∶1 mix of aged cattle blood and decomposed fish oil. We moved traps each session to minimize behavioral response to the lure [36]. For sessions 1–4 in 2006, we also dragged a burlap sack splashed with lure towards game trails in order to increase the attractiveness of the hair trap. For session 5 in 2006 and sessions 1–5 in 2008, we hung a small cloth doused in lure 4–5 m above the center of the trap in order to increase scent dispersion [15]. During session 5 in 2008 we also placed a splash of anise oil on trees within each trap site to attract bears and elicit a rubbing response. We chose hair trap locations before the field season using criteria based on Geographic Information System layers and expert opinion. We selected hair trap locations that were ≥1 km apart and in proximity to seasonal food sources or natural travel corridors. We located each trap ≥100 m from maintained trails, and ≥500 m from heavy human use areas such as campsites. We collected hair samples from each barb separately and also collected discrete clumps of hair below the wire and from the lure pile. We placed samples in paper envelopes labeled with a uniquely numbered barcode and burned every barb after collection to prevent contamination between sessions [15].

We conducted bear rub surveys throughout the study area from mid-August to mid-October 2006, and mid-May to mid-October 2007 and 2008. We focused our efforts on maintained or recently unmaintained trails (<20 years) in the BVSA (Figure 1). We identified 321 bear rubs based on characteristics such as smoothed bark, claw marks, and bear paths [14]. We nailed short pieces of barbed wire to the tree in a zigzag pattern, placed unique numbered ID tags at the back of the base of the tree, and recorded locations with a GPS. Each year we conducted initial surveys on all trails to inspect bear rubs and remove any hair. We then systematically surveyed all trails within the BVSA ≥2 times per year. We collected hair samples from each barb and each wire end separately; we also collected discrete clumps of fresh (i.e. not brittle or bleached) hair from the bark of the tree.

We collected hair from 20 of 25 wildlife crossing structures along the TCH by stretching two lengths of barbed wire perpendicular to the line of movement in order to snag hair from passing bears [38]. Finally, we collected hair opportunistically from various bear management actions, such as during radio collaring efforts and necropsies of human-caused bear mortalities.

### Genetic Analysis

We stored hair samples at room temperature on silica desiccant. Samples were analyzed at Wildlife Genetics International (Nelson, British Columbia), a lab that specializes in analysis of noninvasive genetic samples. The lab used protocols for DNA extraction and microsatellite analysis of samples detailed in Paetkau [39] and validated in Kendall et al. [15]. All DNA extraction was performed using QIAGEN’s DNeasy Tissue Kit (Qiagen, Valencia, CA). We extracted DNA from all samples with ≥1 guard hair follicle or five underfur hairs, using up to 10 guard hairs with underfur when available. We used seven microsatellite loci developed by Paetkau et al. [40] that have become standard for individual identification of grizzly bears in the Rocky Mountains: G10J, G1A, G10B, G1D, G10H, G10M, and G10P.

Mark-recapture estimates can be biased by low power to distinguish individuals (i.e. shadow effect) and genotyping errors such as allelic dropout and false alleles that result in the creation of false individuals [41]–[43]. We therefore used variable markers and genotyping error detection and removal procedures developed by Paetkau [39]. Because we were interested in both species, we chose a procedure that allowed us to obtain black bear results in addition to grizzly without adding substantial costs. For most bear projects in western North America, samples that were extracted are first analyzed at the G10J locus, which has a high amplification success rate and is diagnostic between grizzly and black bears in the region [15]. Instead, we attempted all seven microsatellite loci for all hair samples for which we were able to extract DNA. We excluded samples from further analysis that produced high confidence results for <3 markers. For the samples that produced 3–6 locus genotypes on the first pass, we re-analyzed the markers, but excluded samples from further analysis if they failed on the second pass. Once we had complete seven-locus genotypes, we began the error-checking and removal process by identifying pairs of genotypes that mismatched by one or two loci, a potential warning sign that a genotyping error occurred [39], [44]. We scrutinized the results from any pairs of samples that differed by one or two loci and re-analyzed loci until we had consensus genotypes. To assess the reliability of our genotypes, we used the examining bimodality and difference in capture history tests in program DROPOUT [45]. These tests revealed no problematic loci or samples, allowing us to conclude that our data set was error-free [44], [45]. Once we had complete multi-locus genotypes, we analyzed 1–5 samples from each individual for gender using amelogenin [46], [47]. We used GENALEX [48] to calculate marker power. We calculated expected allele frequencies, expected heterozygosity (H_e_), observed heterozygosity (H_o_), the probability of identity (P_ID_) that two randomly drawn individuals would share the same multi-locus genotype and the probability that full siblings would have identical multi-locus genotypes (P_SIB_)_._

We analyzed all hair samples with adequate genetic material in 2006, but due to budget constraints, we subsampled bear rub samples in 2007 and both bear rub and hair trap samples in 2008. In 2007 and 2008 we genotyped one sample from each bear rub per sampling event (∼50% of samples). For hair traps in 2008, we tried to fit the subselection rules to the budget, with a goal of extracting 50% of the samples.

### Population Abundance

We used data from our three sampling methods to estimate superpopulation abundance of grizzly and black bears in the BVSA during 2006 and 2008. We used hair samples collected from multiple methods (i.e. hair traps, bear rubs, and wildlife crossing structures) to increase sampling coverage and improve precision of abundance estimates [17]. We compiled individual bear encounter histories for each species for each year and analyzed them using Huggins [49] closed population models in program MARK [50], [51].

Our candidate models included sex as a group and hair trap effort (HTE), bear rub effort (BRE; [14], [15]), crossing structure effort (CSE), and distance to edge of grid (DTE; [37]) as individual covariates to model capture probability (*p*) heterogeneity. We created minimum convex polygons (MCP) for each individual using all available location data including data points not used in our abundance estimates from 176 bear rubs outside the study area. We used centroids of MCPs to approximate bear detection centers for 60 grizzly bears and 36 black bears, but for the 20 grizzly bears and 49 black bears which had <3 available points, we took the average of two points or used one point. We calculated DTE as the distance from the edge of the grid to each individual’s detection center. We calculated sex-specific mean maximum distance moved (MMDM) by taking the average of the furthest distance between any two points for each individual with >1 point (black bear F = 8.2 km, M = 13.5 km; grizzly bear F = 14.3 km, M = 23.1 km). We then buffered each individual’s detection center by the MMDM and ½ MMDM. We calculated method-specific sampling effort covariates by summing the number of hair traps (HTE), the cumulative number of days between hair collections for each bear rub tree (BRE), and the number of crossing structures (CSE) that were located within each bear’s idealized detection range during each session. We used Akaike Information Criteria for small samples sizes (AICc) to evaluate relative support for candidate models. We used model averaged estimates of derived parameters to account for model selection uncertainty [52].

We explored whether we could use bear rub-only or hair trap-only data to produce CMR estimates of abundance by comparing them to our models using the combined data set of hair traps, bear rubs, and other methods. We compared abundance estimates for grizzly bears from models using bear rub-only, hair trap-only, and combined data. We compared hair trap-only abundance estimates for black bears with estimates from models using the combined data set.

### Population Trend

We used Pradel [53] open population models to estimate realized population growth rates (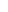) and apparent survival (*φ*) for grizzly bears in the BVSA. We compiled encounter histories using bear rub data collected in 2006, 2007, and 2008. We analyzed encounter histories using robust design models in program MARK by treating years as primary occasions and sampling sessions as secondary occasions [54]. The robust design uses Huggins [49] closed capture models within an open model to estimate abundance for each year as well as annual population growth rates across primary time intervals [54]. We used sex as a group when estimating *p*, 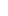, and 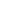. We used BRE and DTE as individual covariates for *p*. We assessed the fit of our models with the median 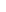 and bootstrap goodness-of-fit tests in MARK.

## Results

### Sampling

We established hair traps from 25 May - 16 August 2006 and 28 May – 18 August 2008. During five 14-day sessions (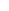 = 13.8 days, SD = 1.0 in 2006, 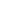 = 14.0 days, SD = 0.7 in 2008), we collected 884 hair samples from 47% of 210 traps in 2006 and 1125 samples from 53% of 210 traps in 2008 (Table 1; Figure 1). We surveyed bear rubs from 10 September - 21 October 2006, 3 June - 27 October 2007, and 28 May – 27 October 2008. We monitored 284 bear rubs in 2006, 321 in 2007, and 313 in 2008. We collected 2910 hair samples from 78% of all bear rubs monitored (n = 321) over the three year period (Table 2; Figure 1). We collected hair samples from 38.4% of rubs in 2006, 59.5% of rubs in 2007, and 56.2% of rubs in 2008. We obtained DNA samples (tissue or hair) from bear management actions and wildlife crossings in the BVSA from 15 May – 18 October 2006, 22 April – 29 October 2007, and 22 April–18 October 2008; we collected 348 samples in 2006, 416 in 2007, and 553 in 2008.

**Table 1:** Bear hair trap results from the Bow Valley of Banff National Park, Alberta, Canada; we conducted hair trapping 25 May – 16 August 2006 and 28 May – 18 August 2008 for five 14-day sessions per year.

								No. Grizzly bears				No. Black bears			
				% traps with	Hair samples/trap^b^		Total no.	New		Unique		New		Unique	
Year	Session^a^	Install dates	No.traps	≥1 hairsample	Mean	SD	hairsamples	F	M	F	M	F	M	F	M
**2006**	1	25 May – 3 Jun	42	59.5	9.7	8.9	243	4	4	4	4	11	9	11	9
	2	7 – 17 Jun	42	52.4	8.0	7.6	177	3	2	4	4	5	0	6	4
	3	21 Jun – 1 Jul	43	37.2	9.1	6.9	146	6	2	9	2	2	1	4	2
	4	5 – 18 Jul	43	41.9	8.9	9.4	160	0	6	3	7	1	1	3	1
	5	19 Jul – 2 Aug	40	45.0	8.8	6.7	158	2	2	3	3	5	5	9	6
Total			210				884			15	16			24	16
Mean			42.0	47.2	8.9	7.9	176.8	3.0	3.2	4.6	4.0	4.8	3.2	6.6	4.4
**2008**	1	28 May – 6 Jun	42	45.2	6.3	5.6	120	3	3	3	3	10	5	10	5
	2	10 – 23 Jun	42	59.5	12.2	12.8	305	2	3	5	3	18	9	21	10
	3	24 Jun – 8 Jul	42	59.5	9.5	8.7	238	10	3	14	5	4	3	10	9
	4	8 Jul – 22 Jul	42	45.2	9.3	9.9	177	2	1	6	4	3	2	11	3
	5	22 Jul – 5 Aug	42	59.5	11.4	10.4	285	0	2	16	4	3	0	8	5
Total			210				1125			17	12			38	19
Mean			42.0	53.8	9.8	9.5	225.0	3.4	2.4	8.8	3.8	7.6	3.8	12.0	6.4

a Hair traps were checked and moved every 13–15 days; 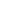 = 13.8 days, SD = 1.0 in 2006 and 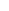 = 14.0 days, SD = 0.7 in 2007.

b Of those hair traps that had ≥1 bear hair sample.

**Table 2:** Bear rub survey results from the Bow Valley of Banff National Park, Alberta, Canada; we conducted bear rub surveys from 10 Sept – 21 Oct 2006, 3 Jun – 27 Oct 2007, and 28 May – 27 Oct 2008.

								No. Grizzly bears				No. Black bears			
				% rubs with	Hair samples/rub^c^		Total no.	New		Unique		New		Unique	
Year	Session^a^	Survey dates	No. rubs^b^	≥1 hair sample	Mean	SD	hair samples	F	M	F	M	F	M	F	M
**2006**	1	10 – 30 Sept	190	35.3	3.7	2.4	249	12	20	12	20	0	1	0	1
	2	1 – 21 Oct	276	18.5	3.1	2.1	159	3	5	6	14	1	0	1	0
Total			284				408			15	25			1	1
Mean			233.0	26.9	3.4	2.3	204.0	7.5	12.5	9.0	17.0	0.5	0.5	0.5	0.5
**2007**	1	3 – 23 Jun	190	40.0	3.5	2.5	265	0	14	0	14	0	0	0	0
	2	24 Jun – 14 Jul	238	34.5	3.1	2.0	253	7	5	7	11	1	2	1	2
	3	15 Jul – 4 Aug	281	23.8	3.4	2.5	231	4	4	5	14	0	4	1	4
	4	5 – 25 Aug	305	19.0	2.7	1.8	154	2	3	4	14	1	0	1	0
	5	26 Aug – 15 Sep	311	13.5	2.4	1.6	102	2	2	7	7	0	2	0	2
	6	16 Sep – 6 Oct	304	17.4	2.3	1.5	122	1	1	5	11	0	1	0	2
	7	7 – 27 Oct	244	12.7	3.0	2.3	94	0	1	2	10	0	0	0	0
Total			321				1221			16	30			2	9
Mean			267.6	23.0	2.9	2.0	174.4	2.3	4.3	4.3	11.6	0.3	1.3	0.4	1.4
**2008**	1	28 May – 22 Jun	116	31.9	5.7	4.1	212	0	7	0	7	0	1	0	1
	2	23 Jun – 22 Jul	213	39.4	4.0	2.8	333	4	7	4	13	2	0	2	1
	3	23 Jul – 23 Aug	259	28.6	3.8	2.4	282	4	4	6	13	0	0	1	1
	4	24 Aug – 13 Sep	285	25.3	3.5	2.4	249	6	6	11	14	2	1	2	2
	5	14 Sep – 4 Oct	281	21.4	2.6	1.6	156	4	0	10	6	0	0	2	2
	6	5 – 27 Oct	221	9.0	2.5	1.5	49	1	0	7	5	0	0	0	0
Total			313				1281			19	24			4	2
Mean			229.2	25.9	3.7	2.5	213.5	3.2	4.0	6.3	9.7	0.7	0.3	1.2	1.2

a Bear rubs were surveyed every 14–21 days.

b Bear rubs were surveyed multiple times per year, therefore the totals do no sum as each bear rub was only counted once per.

session or year.

c Of those bear rub visits that had ≥1 bear hair sample.

### Genetic Analysis

We calculated marker power using data from 80 grizzly bears and 85 black bears identified from hair and tissue samples collected in the Bow Valley. Marker power was high for grizzly and black bears using the same seven loci for individual identification (Table S1). Mean H_o_ across loci was higher for black bears (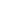 = 0.85, SE = 0.03) than for grizzly bears (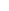 = 0.77, SE = 0.03). The mean number of alleles per locus was also higher for black bears (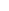 = 10.3, SE = 0.9) than for grizzlies (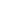 = 7.3, SE = 0.6). P_(ID)_ was 2.3×10^−8^ for grizzly bears and 1.0×10^−9^ for black bears. P_(SIB)_ was 0.0013 for grizzly bears and 0.0007 for black bears (Table S1). We assumed our final multi-locus dataset was error free based on our mismatch distributions, lack of any one or two locus mismatches, and results of our difference in capture history test. As part of another study which is not reported here for the sake of brevity, we ran 13 additional microsatellite loci on each individual which concurred with our original seven-locus genotypes providing further evidence of an error free dataset.

Of the 6236 samples we collected over the three year study, we extracted DNA and attempted genetic analysis on 76.0% (n = 4741). When we analyzed all of the bear rub samples in 2006, we found that we detected >1 individual on a bear rub during a sampling period <5% of the time. Based on these results, in 2007 and 2008, we genotyped one sample from each bear rub per sampling event (∼50% of samples). For hair traps in 2008, we extracted 51.0% of 1125 samples. Of the 3744 samples from bear rubs and hair traps that we extracted and analyzed, only six were identified as non-bear. We obtained individual identities (ID) for 46.4% of all hair trap samples (n = 2009; 51.6% in 2006 and 42.2% in 2008). Of the 2910 bear rub samples, we obtained IDs for 26.8% (n = 779; 62.5% in 2006, 23.0% in 2007, and 19.0% in 2008). Of the 932 samples that produced multi-locus genotypes from hair traps, 43.6% came from black bears (n = 400) and 56.4% came from grizzlies (n = 517). Of the 779 bear rub samples that produced multi-locus genotypes, 94.5% came from grizzly bears (n = 736) and 5.5% came from black bears (n = 43).

We identified a total of 80 unique grizzly bears and 85 black bears from multi-locus genotypes across all methods and years. Genetic analysis of samples collected at hair traps identified 31 grizzly bears and 40 black bears in 2006 and 29 grizzly and 57 black bears in 2008 (Table 1). From bear rub samples, we identified 40 grizzlies and 2 black bears in 2006, 46 grizzlies and 11 black bears in 2007 and 43 grizzlies and 6 black bears in 2008 (Table 2). During the two years that we conducted hair trap and bear rub sampling simultaneously, we identified the majority of individual grizzly bears with bear rub trees, 75.5% in 2006 and 91.5% in 2008, and the majority of black bear individuals with hair traps, 100% in 2006 and 96.6% in 2008 (Table 3). Of the grizzly bears detected at bear rubs and hair traps in 2006, we identified 58% at hair traps; whereas in 2008, we detected 61.7% at hair traps. Only 5% of black bears detected in 2006 were detected with bear rubs and only 10.2% of black bears detected in 2008 were detected with bear rubs. We identified 11 grizzlies at wildlife crossings in 2006, 12 in 2007, and 10 in 2008; we also identified 11 black bears in 2006, 8 in 2007, and 9 in 2008. Of the bears detected at wildlife crossings, we identified some bears that were undetected with hair traps or bear rubs, including: 4 grizzlies in 2006, 3 in 2007, and 1 in 2008, plus 3 black bears in 2006, 7 in 2007, and 4 in 2008.

**Table 3:** Number and percentage of individual grizzly and black bears detected with hair traps and bear rubs in the Bow Valley of Banff National Park, Alberta, Canada in 2006 and 2008.

	2006				2008			
Sampling Method	M		F		M		F	
	**n**	**%**	**n**	**%**	**n**	**%**	**n**	**%**
**Grizzly bears**								
Hair traps-only	6	19.4	7	31.8	1	4.0	3	13.6
Bear rubs-only	15	48.4	7	31.8	13	52.0	5	22.7
Both methods	10	32.3	8	36.4	11	44.0	14	63.6
Total	31		22		25		22	
**Black bears**								
Hair traps-only	15	93.8	23	95.8	18	90.0	35	89.7
Bear rubs-only	0	0.0	0	0.0	1	5.0	1	2.6
Both methods	1	6.3	1	4.2	1	5.0	3	7.7
Total	16		24		20		39	

* Total counts only include bears detected with hair traps or bear rubs, therefore they are <minimum counts which include bears detected with other methods (i.e. wildlife crossings).

### Population Abundance

**Figure 2:**
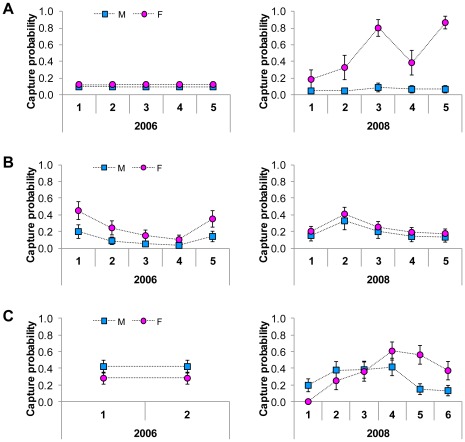
Sex-specific per session capture probability estimates for grizzly and black bears at hair traps and bear rubs in the Bow Valley of Banff National Park, Alberta, Canada. Capture probabilities from (A) grizzly bears at hair traps, (B) black bears at hair traps, and (C) grizzly bears at bear rubs. We derived model-averaged estimates from closed population models for grizzly bears (Table S2) and black bears (Table S3). Error bars represent model averaged estimates of standard error.

**Figure 3:**
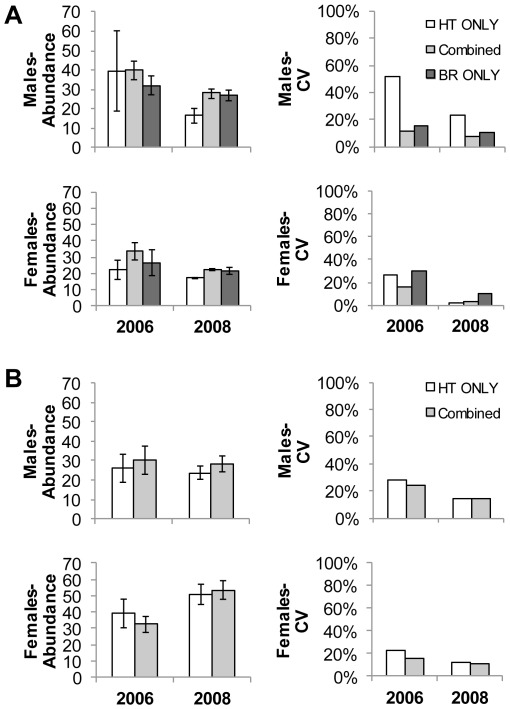
Comparison of Huggins closed population models of abundance for grizzly and black bears in the Bow Valley of Banff National Park, Alberta, Canada. Estimates for (A) grizzly bears and (B) black bears using data from two noninvasive genetic sampling methods, hair traps and bear rubs. Models were created with different datasets, (1) hair trap-only, (2) combined bear rub, and hair trap, and (3) bear rub-only. We constructed hair trap-only and bear rub-only models with the same structure and covariates (DTE, HTE, and BRE) as models using the combined sampling methods. The combined models included data from a third DNA sampling method (i.e. bear management actions, wildlife crossings). We did not create bear rub-only models for black bears due to the low capture probability estimates of black bears at bear rubs. Error bars represent model averaged estimates of standard error.

Our best supported models for grizzly bear abundance allowed capture probabilities to vary by sex in 2006 and sex and time in 2008. Our best supported models contained covariates for DTE, CSE, and BRE in 2006 and DTE, CSE, BRE, and HTE in 2008 (Table S2). We estimated grizzly bear abundance in the BVSA to be 73.5 (95% CI = 64–94) in 2006 and 50.4 (95% CI = 49–59) in 2008 (Table 4). Our estimated per-session hair trap capture probabilities for males (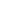 = 0.10, SD<0.01 in 2006; 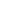 = 0.06, SD = 0.02 in 2008) were lower than females (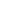 = 0.12, SD<0.01 in 2006; 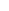 = 0.51, SD = 0.30 in 2008) during all sessions (Figure 2A). Our estimated per-session bear rub capture probabilities for males (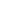 = 0.42, SD<0.01 in 2006; 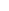 = 0.28, SD = 0.13 in 2008) were higher than females (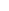 = 0.28, SD = 0.00 in 2006; 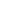 = 0.36, SD = 0.22 in 2008) in 2006, but not in 2008 (Figure 2C). High detection rates of grizzly bears at bear rubs allowed us to produce precise mark-recapture estimates of abundance using bear rub data alone. Abundance estimates for grizzly bears derived from bear rub-only models were more similar to models using combined data from bear rubs, hair traps, and other methods than were hair trap-only estimates (Figure 3A).

**Table 4:** Total minimum counts and model-averaged estimates of grizzly bear abundance in the Bow Valley of Banff National Park, Alberta, Canada, in 2006 and 2008.

					95% CI	
Parameter	Min. Count	Estimate	SE	CV (%)	Lower	Upper
**2006**						
M	32	39.9	4.7	11.9	35	55
F	25	33.6	5.3	15.9	28	51
Pooled	57	73.5	7.2	9.7	64	94
**2008**						
M	26	28.1	2.1	7.6	26	37
F	22	22.3	0.6	2.7	22	26
Pooled	48	50.4	2.2	4.4	49	59

Our best supported models for black bear abundance contained covariates for DTE and HTE and allowed capture probabilities to vary by sex and time in 2006 and 2008 (Table S3). We estimated black bear abundance at 62.6 (95% CI = 51–89) in 2006 and 81.8 (95% CI = 72–102) in 2008 (Table 5). Our estimated per-session hair trap capture probabilities for female black bears (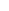 = 0.26, SD = 0.14 in 2006; 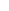 = 0.25, SD = 0.10 in 2008) were higher than males (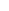 = 0.10, SD = 0.07 in 2006; 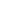 = 0.19, SD = 0.08 in 2008) during all sessions (Figure 2B). Per-session model averaged capture probability estimates for black bears at bear rubs were extremely low so we collapsed sessions into one per year (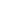 = 0.02, SE = 0.02 for males and females in 2006; 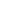 = 0.07, SE = 0.03 for males and females in 2008) in our joint dataset models. Abundance estimates for black bears derived from hair trap-only models were comparable to models using combined data from bear rubs, hair traps, and other methods (Figure 3B).

**Table 5:** Total minimum counts and model-averaged estimates of black bear abundance in the Bow Valley of Banff National Park, Alberta, Canada, in 2006 and 2008.

					95% CI	
Parameter	Min. Count	Estimate	SE	CV (%)	Lower	Upper
**2006**						
M	19	30.2	7.3	24.1	23	55
F	24	32.3	4.9	15.2	27	48
Pooled	43	62.6	9.0	14.4	51	89
**2008**						
M	22	28.3	4.1	14.5	24	42
F	41	53.5	5.7	10.7	46	70
Pooled	63	81.8	7.2	8.8	72	102

* 13 black bear samples from 6 hair traps were not analyzed in 2006.

### Population Trend

**Figure 4:**
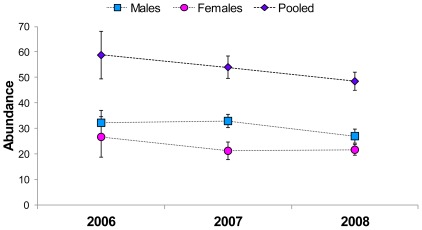
Estimates of abundance derived from Pradel robust design open population models for grizzly bears in the Bow Valley of Banff National Park, Alberta, Canada. Estimates were obtained using three years of bear rub data collected between 2006 and 2008. We derived model averaged estimates from most supported models (Table S4). Error bars represent model averaged estimates of standard error.

Our best supported models for grizzly bear 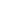 and 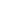 allowed sex-specific capture probabilities to vary with time and contained individual covariates, DTE and BRE, for *p* (Table S4). Time varying models for 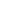 were not considered due to grizzly bear life history and the short study duration. Time-invariant models were constrained to produce a single 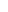 estimate for males and females between the time intervals 2006–2007 and 2007–2008, therefore we only present one 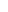 estimate for each sex (Table 6). Our model averaged 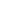 estimates for grizzly bears with three years of bear rub data in the BVSA were 0.93 for males (95% CI = 0.74–1.17) and 0.90 for females (95% CI = 0.67–1.20). Per-session model averaged capture probability estimates from the Pradel [53] model were generally lower for females than for males, except during sessions 2 and 5 in 2007, and 4, 5, and 6 in 2008 (Figure S1). We derived precise abundance estimates (CV<20%, [55]) from robust design models for 2006, 2007, and 2008 (Figure 4). Results of the median 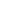
and bootstrap goodness-of-fit tests (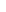
<1) suggested no lack of fit for the general model to our data. We therefore assumed no overdispersion in our data.

**Table 6:** Estimates of realized population growth rate (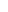) and apparent survival (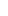) from Pradel open population models for grizzly bears in the Bow Valley of Banff National Park, Alberta, Canada, between 2006 and 2008, sampled using bear rub surveys.

				95% CI	
Parameter	Estimate	SE	CV (%)	Lower	Upper
*λ*					
M	0.93	0.11	12.0	0.74	1.17
F	0.90	0.13	14.9	0.67	1.20
*φ*					
M	0.68	0.07	10.7	0.53	0.81
F	0.77	0.09	11.3	0.56	0.90

* Our models did not vary with time; therefore they produced identical 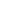 estimates for 2006–2007 and 2007–2008.

## Discussion

Estimating demographic parameters for carnivore populations in protected areas such as BNP is essential for effective management and conservation. We conducted the first detailed evaluation of hair traps and bear rubs for inventorying and monitoring of the sympatric grizzly and black bear populations in BNP. Although studies have reported abundance measures of grizzly and black bears in the Bow Valley of BNP [32], [56], [57], we produced the first rigorous abundance estimates with confidence intervals for grizzly and black bears using DNA-based mark-recapture methods. We demonstrated that bear rubs have higher detection rates than hair traps for grizzly bears and low detection rates for black bears in BNP. We also provided empirical evidence that the grizzly bear population may have declined in the Bow Valley between 2006 and 2008.

### Sampling

Detection rates from the two NGS methods were drastically different for grizzly and black bears in the BVSA (Table 3). Hair traps had high detection rates for grizzly bear females as well as both male and female black bears, but extremely low capture probabilities for male grizzlies (Figure 2A, B). Bear rubs detected very few black bears, but detected many grizzly bears that were not detected at hair traps (Table 3). Competition has been well-documented between grizzly and black bears in North America and is the result of almost complete dietary overlap in some regions [26]. Black bears may avoid bear rubs, or areas with bear rubs, in BNP due to interspecific competition or because rubbing behavior is less advantageous to them compared to grizzlies in the region for some other ecological reason (e.g., fewer ecto-parasites). Limited food resources in BNP resulting in some of the lowest reported grizzly bear densities in North America [19] could explain the relatively high detection rates of grizzlies on bear rubs if rubbing behavior is density dependent. Where grizzly bear density is low and home ranges are large such as in BNP, individual bears could be more detectable with bear rub surveys (i.e. more bear rubs per bear) than in areas with much higher grizzly bear densities and smaller home ranges such as Glacier National Park, MT, USA [14], [15].

### Genetic Analysis

Well-designed sub-sampling can greatly reduce analysis costs while maintaining data integrity by selectively targeting the best samples for extraction and thus the most likely samples to produce individual identities. When we analyzed all of the bear rub samples in 2006, we found that we detected >1 individual on a rub tree during a sampling period <5% of the time so in 2007 and 2008 we subsampled, greatly reducing the number of duplicate samples from the same individuals in time and space while likely missing only a few individuals. For hair traps in 2008, we came very close to our subsampling criteria (50.0%) with an extraction rate of 51.0%.

Genetic analysis was facilitated and expedited by simultaneously analyzing grizzly and black bear samples to obtain individual IDs. The seven microsatellite markers we used to determine individual IDs had high power to distinguish individuals for both grizzly and black bears (Table S1). The proportional cost of obtaining black bear data in addition to grizzly bear data is minor with our analysis approach, especially considering that all time, labor, and equipment costs associated with field collection of hair samples will be paid for (along with DNA extraction) regardless of whether or not individual ID analysis for black bears is performed.

### Population Abundance

Combining bear rubs and hair traps with other DNA sampling methods allowed us to produce precise abundance estimates for grizzly bears (Table 4, CVs<16%) in a relatively small study area with little geographic closure. Our issues with geographic closure were most likely minimized due to our high sampling intensity and the combination of NGS methods providing greater sampling coverage [17], [58]. Our use of DTE and other individual covariates allowed us to model individual capture heterogeneity, resulting in robust estimates of superpopulation abundance [37], [59]. Because our objectives were not to produce density estimates or to correct abundance estimates for closure violation, we chose to present superpopulation abundance estimates to allow for more direct comparisons between sampling methods. Not surprisingly, our estimates of grizzly bear density based on 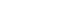 were similar to density estimates based on radio-telemetry data (1.2–1.6 bears/100 km^2^; [56]), but meaningful comparisons are difficult due to differences in study area and methodology.

The improved precision of estimates produced for grizzlies in 2008 compared to 2006 (Table 4) can most likely be explained by a longer bear rub survey season with more sampling sessions coupled with higher capture probabilities at hair traps (Figure 2A). We believe that capture probabilities were higher for grizzly bears at hair traps in 2008 than 2006 due to our greater knowledge of the area based on hair trap success in the first year, our increased understanding of grizzly bear habitat use in the valley, and our more adaptive approach to site selection combined with the use of a more effective lure in the second year. In addition to the measures we described above to increase capture probabilities, during session 5 in 2008 we also used anise oil to further entice bears to enter hair traps which resulted in more female grizzly bears being detected than in any other session (Table 1). As suggested by Stetz et al. [18], liquid scent lures used on bear rub trees could increase capture probabilities and their use should be explored for bear rub surveys.

Our efforts to maximize hair trap detection rates appear to have been successful, but it is remarkable that we achieved such high capture probabilities for female grizzly bears in 2008 and that they were so much higher than in 2006 (Figure 2A). Kendall et al. [15] showed that subadults and dependent offspring are detected with hair traps and bear rubs. In an area like BNP, where the age to independence is 2.5–5.5 years [19] and the seasonal food availability so variable, it is possible that there were more dependent juveniles traveling with their mothers in 2006 than in 2008. Changes in the proportion of females with dependent offspring and/or annual fluctuations in food availability could explain variation in hair trap detection probabilities such as those seen in our study.

Capture probabilities for male grizzlies at hair traps were very low relative to females in 2006 and 2008 (Figure 2A). Other studies have documented higher capture probabilities for females than males at hair traps, but this discrepancy is surprising because research has shown that males typically move greater distances and encounter more hair traps than females [37]. Greater closure violation for males than females could be one explanation for our relatively low male capture probabilities at hair traps. It is also plausible that a high proportion of male grizzly bears in the Bow Valley had already been previously live-captured [56] or encountered hair traps during sampling conducted in large grids to the north and south of the BVSA in 2005 and 2006, respectively [25], and were therefore less likely to enter the enclosure to investigate the lure [37], [60]. Male grizzly bears may also spend less time than females in more human-dominated areas of the Bow Valley such as those found in much of our hair trap sampling grid (Figure 1).

Per session bear rub capture probabilities of grizzly bears were lower for females than males early in the year, but became higher than males as the season progressed (Figure 2C). The same pattern of increasing female capture probabilities has been documented by Kendall et al. [14], [15]. Surprisingly, we were able to obtain abundance estimates from bear rub data alone that were comparable in magnitude and precision to our best estimates using the combined methods data set (Figure 3A). We compared estimates from hair traps and bear rubs because bear population estimates based on hair traps alone have become the convention in much of North America [11], [12]. In general our estimates using bear rub-only data were more accurate and precise than hair trap-only estimates as judged by comparing them to estimates from the combined data set; however, direct comparisons between bear rub-only and hair trap-only estimates are complicated by the difference in sampling duration between methods. The one bear rub-only estimate which was imprecise (CV>20%) was for females in 2006. Based on the capture probabilities we achieved with bear rubs in 2006, we attribute the imprecision of the 2006 estimate for females to a late sampling season and only two bear rub sampling sessions as compared to six sessions in 2008 (Table 2). Our hair trap-only estimates were imprecise for males, especially in 2006, due to low capture probabilities. Our comparison of estimates using different data types supports the findings of other researchers that combining sampling methods increases precision of bear abundance estimates [14], [15], [17], [61]. Our results also suggest that bear rub-only abundance estimates can be comparable to those obtained using both hair traps and bear rubs combined in some grizzly bear populations.

Even though our grid cell size was tailored to the average home range size of grizzly bear females [36], we were able to obtain precise estimates (CV<20%) for black bears in both 2006 and 2008. Bear rubs did not add significantly to the number of black bears we detected in 2006 or 2008 (Table 3). As bear rub capture probabilities were so low for black bears, we attribute the precision of our estimates to the relatively high capture probabilities at hair traps (Figure 2B). Per session capture probabilities at hair traps were higher for females than for males in both years. It appears that there is a decline in capture probabilities through time for both years, but a distinct increase for both males and females in session 5 of 2006. This anomaly could be attributed to hanging a lure-soaked rag as session 5 was the only session where it was used in 2006. Combining bear rubs and hair traps did not improve precision much over estimates for black bears using hair trap-only datasets (Figure 3B), suggesting that wildlife managers interested in monitoring black bears in BNP may need to rely on the use of hair traps alone.

### Population Trend

Using a Pradel [53] open population model with three years of bear rub data, we were able to obtain precise 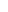 estimates for male and female grizzly bears in the BVSA (Table 6). Our estimates of realized population growth rates (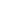 = 0.93, SE = 0.11 for males and 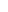 = 0.90, SE = 0.13 for females) and our decreasing annual abundance estimates suggest that the grizzly bear population declined in the BVSA between 2006 and 2008.

We recognize that inferences from our results can only be taken so far with such a small study area and limited time frame, factors which limit our ability to distinguish between emigration, mortality, and low recruitment as the cause(s) of the decline. For example, our 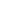 estimates could be driven solely by annual fluctuations in food availability or patterns of reproduction. McCall [62] found that black bear population dynamics in northern Idaho were driven by changes in berry productivity. Similarly, extensive movements of grizzly bears into the Bow Valley in years of good buffaloberry production have been documented [56]. Although annual fluctuations in the abundance of berries could influence our results, an examination of berry productivity data collected in BNP between 2006 and 2010 [63] shows no correlation between berry production and the number of bears detected in the BVSA. Our 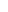 estimates could also reflect the dynamics of a population returning to a more stable size for the Bow Valley after a spike in reproduction (i.e. birth pulse). Regardless of the limitations to identifying drivers for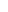, an undeniable downward population trend exists in bear rub-only abundance estimates for male and female grizzlies across the three years of our study (Figure 4). This trend was also apparent in the abundance estimates for 2006 and 2008 from closed population models using combined bear rub/hair trap data (Table 4) and hair trap-only data (Figure 3A).

Although our objective of estimating 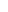 for grizzly bears using open population models was to examine the feasibility of using bear rub data for long-term population trend monitoring in BNP, we were able to obtain empirical evidence suggesting that the grizzly bear population declined in the Bow Valley during our study. Between 1994 and 2002, Garshelis et al. [19] monitored radio-collared grizzly bears in BNP and determined that the population was slightly increasing while also being one of the slowest reproducing grizzly bear populations yet studied at the time of their research. They estimated projected population growth rates for grizzly bears (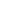 = 1.04, 95% CI = 0.99–1.09) using Leslie matrices, but concluded that the population would have declined if adult female mortality had increased during their study. Other studies have also indicated that grizzly bear population growth rates are most sensitive to changes in adult female survival [64]–[66]; therefore, the decline we detected was not surprising as adult female mortality rates exceeded BNP’s established threshold between 2002 and 2008 [67]. In fact, known human-caused independent female mortality rates more than doubled after Garshelis et al.’s [19] study ended [67]. Fewer adult females along with the accompanied reduction in births could explain the decline we detected in our study area even if emigration, mortality and immigration were constant. Whatever the driver(s) may be, our 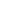 estimates show concordance with previous research suggesting that the Bow Valley may act as an attractive sink for grizzly bears in the Central Canadian Rocky Mountains [68]–[70], but these findings should be confirmed with long-term monitoring across a much larger geographic area.

Our results concur with previous research [71], [72] demonstrating that DNA-based mark-recapture methods can produce precise estimates of population growth rates in relatively short periods of time compared to radio-collar (i.e. known fate) methods, therefore reducing some of the uncertainty when managing threatened bear populations. We have shown that bear rub surveys in BNP can be efficient through subsampling to reduce analysis costs and by partnering with existing Parks Canada staff and their Citizen Scientist volunteer program to reduce labor costs. Bear rub surveys also require no lure, therefore have less behavioral response issues and fewer public safety concerns associated with them compared to hair traps. These characteristics make bear rubs particularly well-suited for long-term grizzly bear trend monitoring programs in human-dominated landscapes such as BNP; however, we recommend that the spatial and temporal scale of future bear rub surveys be extended as much as possible to avoid issues of closure violation, to determine long-term population trend, and to be able to link changes in demographics to ultimate drivers of population abundance and growth in the region. We conclude that bear rub surveys provide wildlife managers with an efficient, reliable technique to inventory and monitor grizzly bear populations in the Central Canadian Rocky Mountains.

## Supporting Information

Figure S1 Sex-specific per session capture probability estimates from Pradel robust design open population models. Estimates obtained using bear rub data collected in the Bow Valley of Banff National Park, Alberta, Canada, between 2006 and 2008. We derived model averaged capture probability estimates from most supported models (Table S4). Bear rub effort (BRE) was the cumulative number of days between successive hair collections summed over all bear rubs sampled per session: values were divided by 10,000 to standardize to scale of *y* axis. Error bars represent model averaged estimates of standard error.
(TIF)

Table S1 Microsatellite marker variability for determining individual identity of grizzly (n = 80) and black bears (n = 85) from DNA samples collected from hair traps, bear rubs, wildlife crossing structures, and bear management actions in the Bow Valley of Banff National Park, Alberta, Canada between May 2006 and October 2008.
(PDF)

Table S2 Model selection results from Huggins closed population models of grizzly bear abundance in the Bow Valley of Banff National Park, Alberta, Canada in 2006 and 2008; results from Program MARK, April 2011 build.
(PDF)

Table S3 Model selection results from Huggins closed population models of black bear abundance in the Bow Valley of Banff National Park, Alberta, Canada in 2006 and 2008; results from Program MARK, April 2011 build.
(PDF)

Table S4Model selection results from Pradel open population models of realized population growth rate (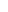) and apparent survival (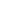) for grizzly bears in the Bow Valley of Banff National Park, Alberta, Canada between 2006 and 2008; results from Program MARK, April 2011 build.(PDF)
