# Correction: Estimating Grizzly and Black Bear Population Abundance and Trend in Banff National Park Using Noninvasive Genetic Sampling

**DOI:** 10.1371/annotation/47e6e4a4-006e-4423-8b78-85d405e97333

**Published:** 2012-05-31

**Authors:** Michael A. Sawaya, Jeffrey B. Stetz, Anthony P. Clevenger, Michael L. Gibeau, Steven T. Kalinowski

There is a grammatical error in the Funding section. It should read: This project was generously supported by Parks Canada, the Western Transportation Institute-Montana State University (WTI), the Woodcock Foundation, the Henry P. Kendall Foundation, and the Wilburforce Foundation. Other support was provided by the National Fish and Wildlife Foundation, Alberta Conservation Association, Calgary Foundation, and the Mountain Equipment Cooperative. The funders had no role in study design, data collection and analysis, decision to publish, or preparation of the manuscript.

