# Supplementary figures and images for: Estimating Grizzly and Black Bear Population Abundance and Trend in Banff National Park Using Noninvasive Genetic Sampling
Source: PLoS One. 2012 May 2;7(5):e34777. doi: 10.1371/journal.pone.0034777 (PMC3342321; doi:10.1371/journal.pone.0034777)

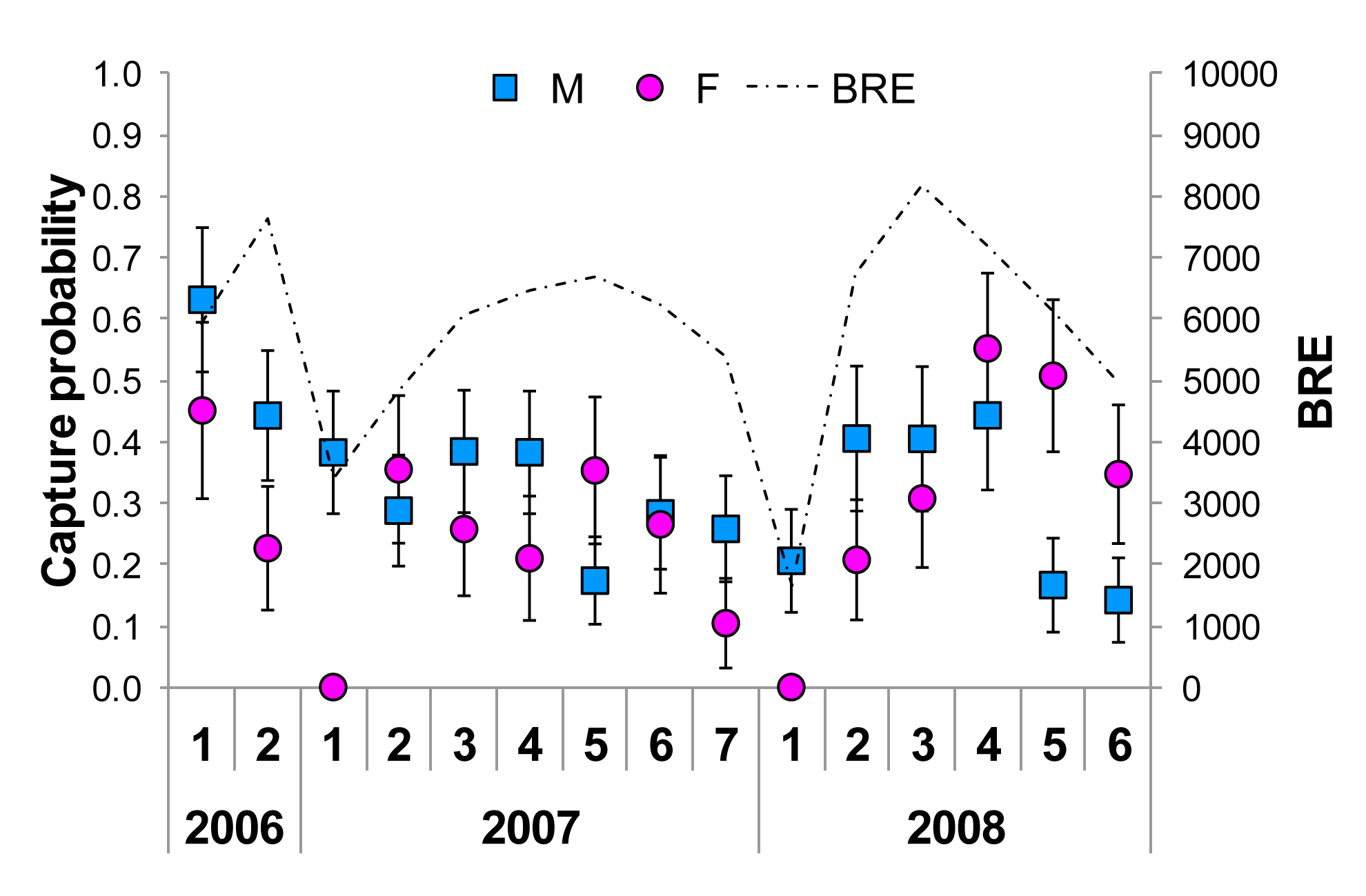

Supplement: Figure S1 — Sex-specific per session capture probability estimates from Pradel robust design open population models. Estimates obtained using bear rub data collected in the Bow Valley of Banff National Park, Alberta, Canada, between 2006 and 2008. We derived model averaged capture probability estimates from most supported models (Table S4). Bear rub effort (BRE) was the cumulative number of days between successive hair collections summed over all bear rubs sampled per session: values were divided by 10,000 to standardize to scale of y axis. Error bars represent model averaged estimates of standard error. (TIF) [file pone.0034777.s001.tif]
