# Supplementary material for: Estimating Grizzly and Black Bear Population Abundance and Trend in Banff National Park Using Noninvasive Genetic Sampling
Source: PLoS One. 2012 May 2;7(5):e34777. doi: 10.1371/journal.pone.0034777 (PMC3342321; doi:10.1371/journal.pone.0034777)
Supplement: Table S1 — Microsatellite marker variability for determining individual identity of grizzly (n = 80) and black bears (n = 85) from DNA samples collected from hair traps, bear rubs, wildlife crossing structures, and bear management actions in the Bow Valley of Banff National Park, Alberta, Canada between May 2006 and October 2008. (PDF) [file pone.0034777.s002.pdf]

Table S1. Microsatellite marker variability for determining individual identity of grizzly (n=80) and black bears (n=85) from DNA samples collected from hair traps, bear rubs, wildlife crossing structures, and management actions in the Bow Valley of Banff National Park, Alberta, Canada between May 2006 and October 2008.

	**Grizzly bears**						**Black bears**				
**Marker**	**H_E**	**H_O**	**A**	**P_{ID}**	**P_{SIB}**		**H_E**	**H_O**	**A**	**P_{ID}**	**P_{SIB}**
G1A	0.70	0.69	6	0.08	0.38		0.76	0.73	12	0.04	0.33
G1D	0.82	0.85	10	0.13	0.43		0.81	0.82	9	0.09	0.39
G10B	0.83	0.81	9	0.05	0.35		0.80	0.78	7	0.07	0.37
G10H	0.80	0.80	8	0.06	0.35		0.88	0.94	10	0.06	0.36
G10J	0.79	0.80	6	0.06	0.37		0.85	0.87	14	0.02	0.32
G10M	0.75	0.78	6	0.10	0.40		0.82	0.94	11	0.06	0.36
G10P	0.70	0.65	6	0.13	0.43		0.84	0.84	9	0.05	0.34
Mean	0.77	0.77	7.3				0.82	0.85	10.3		
SE	0.02	0.03	0.6				0.02	0.03	0.9		
Overall probability											
of identity				2.30E-08	0.0013					1.00E-09	0.0007

*H_E=expected heterozygosity; H_O=observed heterozygosity; A=no. of alleles; P_{ID}=probability of identity; P_{SIB}=probability of sibling identity.
