# Supplementary material for: Estimating Grizzly and Black Bear Population Abundance and Trend in Banff National Park Using Noninvasive Genetic Sampling
Source: PLoS One. 2012 May 2;7(5):e34777. doi: 10.1371/journal.pone.0034777 (PMC3342321; doi:10.1371/journal.pone.0034777)
Supplement: Table S2 — Model selection results from Huggins closed population models of grizzly bear abundance in the Bow Valley of Banff National Park, Alberta, Canada in 2006 and 2008; results from Program MARK, April 2011 build. (PDF) [file pone.0034777.s003.pdf]

Table S2. Model selection results from Huggins closed population models of grizzly bear abundance in the Bow Valley of Banff National Park, Alberta, Canada in 2006 and 2008; results from Program MARK, April 2011 build.

Model[a]	AIC_c[b]	ΔAIC_c[c]	w_i[d]	Model Likelihood	No. parameters	Deviance
**2006**						
CS: p(CSE_{half} × DTE) HT: p(sex × DTE) BR: p(sex + BRE_{half}); MF5km	395.11	0.00	0.39	1.00	9	376.70
CS: p(CSE_{half} × DTE) HT: p(sex × DTE) BR: p(sex); M10km, F5km	395.74	0.63	0.28	0.73	8	379.42
CS: p(CSE_{half} × DTE) HT: p(sex × DTE) BR: p(sex); MF5km	395.74	0.63	0.28	0.73	8	379.42
CS: p(CSE_{half} × DTE) HT: p(sex × DTE) BR: p(sex × DTE)	400.97	5.86	0.02	0.05	8	384.65
CS: p(CSE_{half} + DTE) HT: p(sex + DTE) BR: p(sex + DTE)	401.72	6.62	0.01	0.04	7	387.47
CS: p(CSE_{half} × DTE) HT: p(sex × DTE) BR: p(sex × DTE); M10km, F5km	403.77	8.67	0.01	0.01	8	387.45
CS: p(CSE_{half} × DTE) HT: p(sex × DTE) BR: p(sex × DTE); MF5km	403.77	8.67	0.01	0.01	8	387.45
CS: p(CSE_{half}) HT: p(sex) BR: p(sex)	406.25	11.15	0.00	0.00	6	394.07
CS: p(CSE_{full} + DTE) HT: p(sex + DTE) BR: p(sex + DTE)	418.62	23.51	0.00	0.00	7	404.37
CS: p(DTE) HT: p(sex × DTE) BR: p(sex); M10km, F5km	419.06	23.95	0.00	0.00	7	404.81
**2008**						
CS: p(CSE_{half} × DTE) HT: p(sex × time + HTE_{half} × DTE) BR: p(sex × time + BRE_{half}); MF5km	599.93	0.00	0.17	1.00	27	543.17
CS: p(CSE_{half} × DTE) HT: p(sex × time + HTE_{half} × DTE) BR: p(sex × time + BRE_{half}); M10km, F5km	599.93	0.00	0.17	1.00	27	543.17
CS: p(CSE_{half} × DTE) HT: p(sex × time + HTE_{half} × DTE) BR: p(sex × time + BRE_{half}); MF10km	599.93	0.00	0.17	1.00	27	543.17
CS: p(CSE_{half} × DTE) HT: p(sex × time + HTE_{half} × DTE) BR: p(sex × time + BRE_{half})	599.93	0.00	0.17	1.00	27	543.17
CS: p(CSE_{half} + DTE) HT: p(sex × time + HTE_{full}+ DTE) BR: p(sex × time + BRE_{full} + DTE)	601.63	1.70	0.07	0.43	26	547.07
CS: p(CSE_{half} + DTE) HT: p(sex × time + HTE_{half} + DTE) BR: p(sex × time + BRE_{full})	602.16	2.23	0.06	0.33	26	547.60
CS: p(CSE_{half}) HT: p(sex × time + HTE_{half}) BR: p(sex × time + BRE_{half})	603.98	4.04	0.02	0.13	25	551.61
CS: p(CSE_{half} × DTE) HT: p(sex × time × DTE) BR: p(sex × time + BRE_{half}); MF5km	604.19	4.26	0.02	0.12	26	549.63
CS: p(CSE_{half} × DTE) HT: p(sex × time + HTE_{half} × DTE) BR: p(sex × time + BRE_{half} × DTE); MF5km	604.30	4.37	0.02	0.11	27	547.54
CS: p(CSE_{half} × DTE) HT: p(sex × time + HTE_{half} × DTE) BR: p(sex × time + BRE_{half} × DTE); M10km, F5km	604.30	4.37	0.02	0.11	27	547.54

[a] Models estimated capture probabilities, *p*, for three noninvasive genetic sampling methods (HT=hair traps, BR=bear rubs, CS=crossing structures) and contained method-specific sampling effort covariates calculated by summing the number of hair traps (HTE), bear rub days (BRE), and crossing structures (CSE) that were located within each bear's idealized detection range each session. Models also included distance to edge of grid (DTE) covariates; thresholds for DTE, if any, appear at the end of model names after a semicolon (M=males, F=females; km=kilometers).

[b] Akaike's Information Criterion for small sample sizes.

[c] The difference in AIC_c value between the *i*th model and the model with the lowest AIC_c value.

[d] Akaike wt used in model averaging.
