# Supplementary material for: Estimating Grizzly and Black Bear Population Abundance and Trend in Banff National Park Using Noninvasive Genetic Sampling
Source: PLoS One. 2012 May 2;7(5):e34777. doi: 10.1371/journal.pone.0034777 (PMC3342321; doi:10.1371/journal.pone.0034777)
Supplement: Table S4 — Model selection results from Pradel open population models of realized population growth rate () and apparent survival () for grizzly bears in the Bow Valley of Banff National Park, Alberta, Canada between 2006 and 2008; results from Program MARK, April 2011 build. (PDF) [file pone.0034777.s005.pdf]

Table S4. Model selection results from Pradel open population models of realized population growth rate (λ) and apparent survival (φ) for grizzly bears in the Bow Valley of Banff National Park, Alberta, Canada between 2006 and 2008; results from Program MARK, April 2011 build.

Model[a]	AIC_c[b]	ΔAIC_c[c]	w_i[d]	Model Likelihood	No. parameters	Deviance
φ(sex) λ(sex) p(t × sex + BRE_{full} + DTE)	1079.53	0.00	0.36	1.00	36	995.53
φ(sex) λ(sex) p(t × sex + BRE_{full} + DTE); MF10km	1079.53	0.00	0.36	1.00	36	995.53
φ(sex) λ(sex) p(t × sex + BRE_{half} + DTE)	1083.12	3.59	0.06	0.17	36	999.12
φ(sex) λ(sex) p(t × sex + DTE)	1083.46	3.93	0.05	0.14	33	1007.49
φ(sex) λ(sex) p(t × sex + DTE); MF10km	1083.46	3.93	0.05	0.14	33	1007.49
φ(sex) λ(sex) p(t × sex + DTE); M10km, F5km	1083.46	3.93	0.05	0.14	33	1007.49
φ(sex) λ(sex) p(t × sex + DTE); MF5km	1083.46	3.93	0.05	0.14	33	1007.49
φ(sex) λ(sex) p(t × sex × DTE)	1086.09	6.56	0.01	0.04	34	1007.46
φ(sex) λ(sex) p(t × sex × DTE); M10km, F5km	1086.09	6.56	0.01	0.04	34	1007.46
φ(sex) λ(sex) p(t × sex)	1095.30	15.77	0.00	0.00	32	1021.96

[a] Models estimated sex-specific parameters, φ, λ, and bear rub capture probabilities (*p*). Most models contained a covariate for the distance from each bear's detection center to the edge of grid (DTE); thresholds for DTE, if any, appear at the end of model names after a semicolon (M=males, F=females; km=kilometers). Some models contained an individual covariate for the number of bear rub days (BRE) located within each bear's idealized detection range each session. Parameter estimates derived from models with no covariates were similar to estimates from models with covariates for *p*.

[b] Akaike's Information Criterion for small sample sizes.

[c] The difference in AIC_c value between the *i*th model and the model with the lowest AIC_c value.

[d] Akaike wt used in model averaging.
